# Draft Genome Sequence of *Brucella abortus* Virulent Strain 544

**DOI:** 10.1128/genomeA.00419-15

**Published:** 2015-05-07

**Authors:** D. K. Singh, Ashok Kumar, A. K. Tiwari, Jagadesan Sankarasubramanian, Udayakumar S. Vishnu, Jayavel Sridhar, Paramasamy Gunasekaran, Jeyaprakash Rajendhran

**Affiliations:** a Division of Veterinary Public Health, Indian Veterinary Research Institute, Izatnagar, Uttar Pradesh, India; b Division of Biological Standardization, Indian Veterinary Research Institute, Izatnagar, Uttar Pradesh, India; c Department of Genetics, School of Biological Sciences, Madurai Kamaraj University, Madurai, Tamil Nadu, India

## Abstract

Here, we present the draft genome sequence and annotation of *Brucella abortus* virulent strain 544. The genome of this strain is 3,289,405 bp long, with 57.2% G+C content. A total of 3,259 protein-coding genes and 60 RNA genes were predicted.

## GENOME ANNOUNCEMENT

*Brucella* spp. are Gram-negative facultative intracellular bacteria that cause a zoonosis called brucellosis (1). They are nonmotile and non-spore-forming coccobacilli belonging to the *Alphaproteobacteria*. *Brucella* spp. infect a wide range of animals, from dolphins and domestic animals to humans (2). The common bacterial virulence factors, such as exotoxins, pili, fimbriae, cytolysins, flagella, virulence plasmids, etc., are completely missing in *Brucella* species (3). *Brucella abortus* 544 is the most commonly used strain for experimental challenge in animals vaccinated with live or killed *B. abortus* vaccines (4). Here, we present the draft genome sequence of *B. abortus* 544 and its annotation.

The genomic DNA of *B. abortus* 544 was isolated using the DNeasy kit (Qiagen, Hilden, Germany). The 16S rRNA sequence of this strain showed 100% similarity with that of all other *Brucella* species. Therefore, species differentiation was performed by multilocus sequence analysis (MLSA) with 9 loci, as previously described (5). The genome was sequenced using the Ion Torrent personal genome machine (Life Technologies, Carlsbad, CA). In total, 2,736,169 reads, with an average read length of 217 bp, were obtained, which yielded 618.46 Mb of total sequenced bases with 187× fold coverage. The *de novo* assembly was performed using Mimicking Intelligent Read Assembly (MIRA) version 3.9.18 (6), which yielded 33 contigs. The largest contig was 329,558 bp long. The genome size of *B. abortus* strain 544 was 3,289,405 bp (3.28 Mb), with a G+C content of 57.2%. The genome sequence was annotated using the Rapid Annotations using Subsystems Technology (RAST) server (7) and the NCBI Prokaryotic Genome Annotation Process (http://www.ncbi.nlm.nih.gov/genome/annotation_prok/). A total of 3,319 genes were predicted. Of these predicted genes, 3,259 were protein-coding genes. Overall, 2,576 of the protein-coding genes were assigned putative functions, and 683 genes were annotated as hypothetical proteins. rRNA and tRNA genes were predicted using RNAmmer (8) and tRNAscan-SE 1.21 (9), respectively. A total of 60 RNA genes were predicted, of which 9 are rRNA genes and 51 are tRNA genes. *B. abortus* bv. 1 strain NI435a and *B. abortus* bv. 1 strain NI474 were identified as the closest neighbors of *B. abortus* 544 using RAST annotation.

Genes involved in virulence, disease, and the defense mechanisms of the bacterium were identified. These include genes responsible for antibiotic and toxic compound resistance, intracellular survival, invasion, among others. In-depth comparative analysis with other virulent strains and vaccine strains of *B. abortus* will help understand the pathogenic mechanism and intracellular life of *Brucella* organisms.

### Nucleotide sequence accession numbers.

This whole-genome shotgun project has been deposited at DDBJ/EMBL/GenBank under the accession no. JPHK00000000. The version described in this paper is version JPHK01000000.
